# Supplementary material for: The microbiome of the lichen Lobaria pulmonaria varies according to climate on a subcontinental scale
Source: Environ Microbiol Rep. 2024 Jun 23;16(3):e13289. doi: 10.1111/1758-2229.13289 (PMC11194104; doi:10.1111/1758-2229.13289)
Supplement: Supplementary file 1 — Table S1. Families of NCBI taxonomy database included in the custom database used in this study. Figure S1. 10%, 20% and 30% missing values were introduced and imputed using mean, median, random forest (RF), singular value decomposition (SVD) and k‐nearest neighbours (kNN) methods. Performance of the imputation methods was evaluated based on normalized root mean squared error (NRMSE) and NRMSE‐based sum of ranks. Table S2. Symbiont‐specific SSR markers. Number of alleles, allele size range and percent of missing data detected for the 46 investigated samples are given. Table S3. Results of the SSR analysis for both symbiotic partners of Lobaria pulmonaria for the 12 investigated localities. Given are the unique multilocus haplotypes for the fungal and algal partner (uMLHf and uMLHa), the resulting genetic clusters from the cluster analysis for the two partners (GCf and GCa) and the corresponding climate region. [file EMI4-16-e13289-s001.docx]

**The microbiome of the lichen *Lobaria pulmonaria* varies according to climate on a sub-continental scale**

**List of Supplementary Tables and Figures**

Supplementary Table 1: Families of NCBI taxonomy database included in the custom database used in this study.


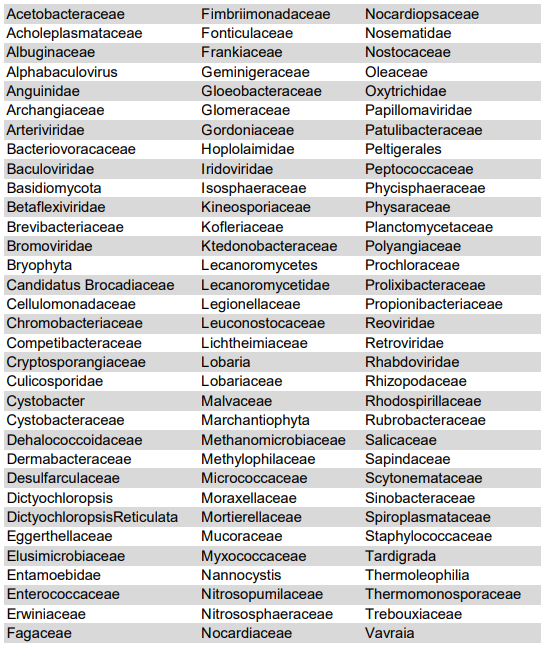


Supplementary Figure 1: 10%, 20%, and 30% missing values were introduced and imputed using mean, median, random forest (RF), singular value decomposition (SVD), and k-nearest neighbors (kNN) methods. Performance of the imputation methods was evaluated based on normalized root mean squared error (NRMSE) and NRMSE-based sum of ranks.


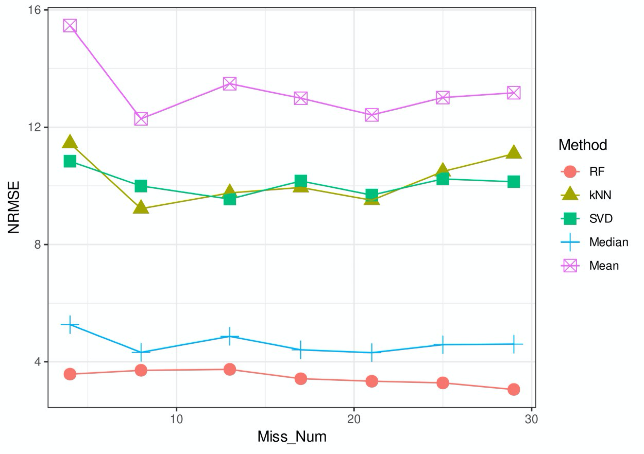

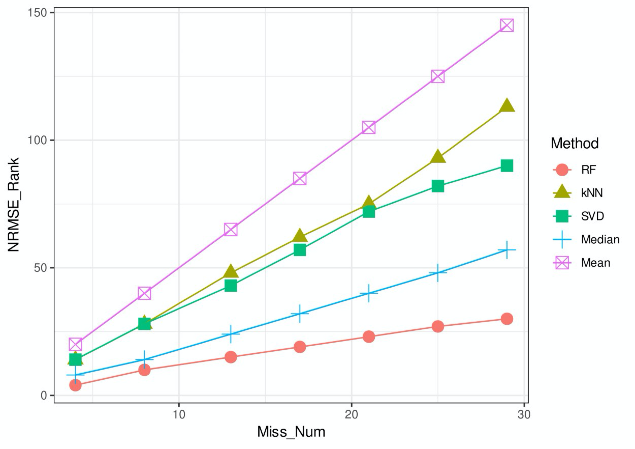


Supplementary Table 2: Symbiont-specific SSR markers. Number of alleles, allele size range and percent of missing data detected for the 46 investigated samples are given.

|  | Number of alleles | Allele size range (bp) | % missing |
| --- | --- | --- | --- |
| Fungus-specific SSR markers | | |  |
| LPu03 | 4 | 185-191 | 2.2 |
| LPu09 | 4 | 173-281 | 6.5 |
| LPu15 | 8 | 157-199 | 0.0 |
| LPu23 | 3 | 295-312 | 0.0 |
| LPu24 | 2 | 229-239 | 0.0 |
| LPu25_12 | 16 | 196-292 | 0.0 |
| LPu28 | 11 | 270-326 | 0.0 |
| MS4 | 3 | 184-238 | 0.0 |
| Algae-specific SSR markers | | |  |
| 6816 | 9 | 160-231 | 0.0 |
| 6819 | 11 | 136-173 | 4.3 |
| 6820 | 7 | 197-214 | 0.0 |
| 6825_2 | 3 | 133-153 | 0.0 |
| 6528 | 4 | 102-108 | 0.0 |
| 6861_3 | 3 | 120-127 | 0.0 |
| 6863 | 7 | 147-198 | 0.0 |
| 7000_2 | 5 | 155-164 | 0.0 |
| 7007_2 | 5 | 171-179 | 0.0 |
| LPu16 | 9 | 196-212 | 0.0 |
| LPu19 | 2 | 441-444 | 30.4 |
| LPu20 | 12 | 190-254 | 0.0 |
| LPu26 | 20 | 354-513 | 23.9 |
| LPu27 | 3 | 194-198 | 0.0 |

Supplementary Table 3: Results of the SSR analysis for both symbiotic partners of *Lobaria pulmonaria* for the 12 investigated localities. Given are the unique multilocus haplotypes for the fungal and algal partner (uMLHf and uMLHa), the resulting genetic clusters from the cluster analysis for the two partners (GCf and GCa) and the corresponding climate region.

| Location | uMLHf | uMLHa | GCf | GCa | Climate Region* |
| --- | --- | --- | --- | --- | --- |
| Stara Rzeka1 | f1 | a1 | fA | aA | SCL |
| Stara Rzeka2 | f1 | a1 | fA | aA | SCL |
| Stara Rzeka3 | NA | NA | NA | NA | SCL |
| Rhön1 | f2 | a2 | fB | aA | HM |
| Rhön2 | f3 | a3 | fB | aB | HM |
| Rhön3 | f4 | a4 | fA | aB | HM |
| Darß | f5 | a5 | fB | aA | SAL |
| Darß | f5 | a5 | fB | aA | SAL |
| Darß | f5 | a5 | fB | aA | SAL |
| Darß | f5 | a5 | fB | aA | SAL |
| Darß | f6 | a6 | fC | aA | SAL |
| Darß | f6 | a6 | fC | aA | SAL |
| Darß | f6 | a7 | fC | aA | SAL |
| Darß | f7 | a8 | fC | aC | SAL |
| Darß | f7 | a8 | fC | aC | SAL |
| Darß | f7 | a9 | fC | aC | SAL |
| Lihme1 | f8 | a10 | fC | aC | SAL |
| Lihme2 | f9 | a10 | fC | aC | SAL |
| Lihme3 | f9 | a11 | fC | aC | SAL |
| Rebild1 | f10 | a12 | fB | aC | SAL |
| Rebild2 | f10 | a12 | fB | aC | SAL |
| Rebild3 | f10 | a13 | fB | aC | SAL |
| Rold Skov1 | f11 | a14 | fA | aC | SAL |
| Rold Skov2 | f11 | a14 | fA | aC | SAL |
| Rold Skov3 | f11 | a14 | fA | aC | SAL |
| Viborg1 | f12 | a15 | fC | aC | SAL |
| Viborg2 | f12 | a16 | fC | aC | SAL |
| Viborg3 | f12 | a15 | fC | aC | SAL |
| Johannishus1 | f13 | a17 | fA | aA | SAL |
| Johannishus2 | f14 | a18 | fA | aA | SAL |
| Johannishus3 | f14 | a17 | fA | aA | SAL |
| Kullen1 | f15 | a19 | fC | aC | SAL |
| Kullen2 | f16 | a20 | fC | aC | SAL |
| Kullen3 | f15 | a21 | fC | aC | SAL |
| Söderåsen | f17 | a22 | fC | aB | SAL |
| Söderåsen | f17 | a23 | fC | aB | SAL |
| Söderåsen | f17 | a24 | fC | aB | SAL |
| Söderåsen | f17 | a25 | fC | aB | SAL |
| Söderåsen | f17 | a26 | fC | aB | SAL |
| Stensnäs | f18 | a27 | fA | aC | SAL |
| Stensnäs | f18 | a28 | fA | aC | SAL |
| Stensnäs | f18 | a28 | fA | aC | SAL |
| Stensnäs | f18 | a29 | fA | aC | SAL |
| Stensnäs | f18 | a28 | fA | aC | SAL |
| Vånga1 | f19 | a30 | fB | aA | SAL |
| Vånga2 | f19 | a30 | fB | aA | SAL |
| Vånga3 | f19 | a30 | fB | aA | SAL |

HM – Hercynian Montane, SAL – Sub Atlantic Lowland, SCL – Sub Continental Lowland.
